# The quality and reliability of short videos on acute myeloid leukemia on Bilibili and TikTok: A cross-sectional study

**DOI:** 10.1097/MD.0000000000047594

**Published:** 2026-02-13

**Authors:** Siqin Yang, Yuqing Li, Jun Tao

**Affiliations:** a Medical Center of Hematology, Xinqiao Hospital, State Key Laboratory of Trauma, Burn and Combined Injury, Army Medical University, Chongqing, China.

**Keywords:** acute myeloid leukemia, Bilibili, information quality, public health, short video, social media, TikTok

## Abstract

Acute myeloid leukemia (AML) is a life-threatening hematological malignancy. With the rapid development of short video platforms, TikTok and Bilibili have become important sources of health information; however, the quality and reliability of AML-related content remain unclear. This study aims to evaluate the content, quality, and reliability of short AML videos on these platforms. The top 150 AML-related videos were collected from each platform using default ranking. Video quality was assessed using 3 validated instruments: the global quality score, modified DISCERN, and the *Journal of American Medical Association* benchmark. The correlations between user engagement metrics (likes, comments, shares, and favorites) and quality scores were also analyzed. In total, 176 videos were included. Most videos focused on treatment (TikTok and Bilibili: 31.6% and 36.1%, respectively) and prognosis (TikTok and Bilibili: 24.4% vs 17.3%, respectively), while pathogeny and clinical manifestations were insufficiently covered. The overall quality was modest: global quality score median 3.00 (interquartile range [IQR]: 2.00–4.00), modified DISCERN median 2.00 (IQR: 1.00–3.00), and *Journal of American Medical Association* median 2.00 (IQR: 2.00–3.00). TikTok videos demonstrated a significantly higher engagement than Bilibili videos (*P* < .05), whereas Bilibili videos were longer (*P* < .05). Videos uploaded by hematologists received the highest scores across all 3 tools (all *P* < .001) but showed relatively low user engagement. No correlation was found between engagement metrics and quality scores (*P* > .05). Short video platforms have become an important source of AML information; however, their overall content quality is limited. Videos created by hematologists are the most reliable; however, user engagement does not reflect information quality. Professional physicians should be encouraged to actively participate in science communication, and platform regulations and algorithm optimization should be strengthened to promote the dissemination of high-quality information.

## 1. Introduction

Acute myeloid leukemia (AML) is a clonal hematopoietic cancer that disrupts normal hematopoietic function and eventually leads to bone marrow failure and death.^[1]^ The common symptoms are anemia, bleeding, infection, hepatosplenomegaly, weakness, fever, etc.^[2,3]^ Chemotherapy is the primary treatment for AML. Remission induction plays an auxiliary role in chemotherapy, that is, by using a standard or high dose of chemotherapy drugs to reduce the number of cancer cells in the patient’s body. The primary goal of this phase is to achieve complete remission (CR).^[4]^ Intensive and repeated chemotherapy, while potentially curative, imposes a significant burden of toxicity that severely compromises the quality of life of AML patients. This treatment leads to a range of debilitating physical symptoms such as fatigue, infections, and nutritional issues, as well as profound psychological distress, including anxiety, depression, and cognitive impairment.^[5]^ In addition, AML is under considerable physical and psychological pressure at the time of diagnosis and before chemotherapy. Social support as an external resource can positively impact the health of individuals under stressful life conditions. A number of studies have shown that mental and physical fatigue in cancer patients are related to less perceived social support, and that good social support can reduce cancer-related fatigue.^[6,7]^ The dissemination of the etiology, diagnosis, treatment, and latest progress of AML through various channels helps patients to understand AML deeply, relieve their anxiety and worry, and strengthen their confidence in resisting the disease.

Short video platforms have become an influential medium for disseminating health information, leveraging multimodal integration, and combining visual, auditory, and textual elements to enhance public comprehension and engagement with complex medical topics. Their format supports efficient communication, improves information retention, and increases accessibility for diverse audiences. Empirical evidence suggests that video-based narratives are particularly effective in knowledge dissemination, often surpassing text-based formats in both reach and impact.^[8]^ The widespread adoption of such platforms is reflected in user statistics. As of 2024, short video users in China accounted for 96.6% of the total Internet population, amounting to 1.07 billion individuals.^[9]^ Globally, platforms, such as TikTok and Bilibili, have substantial user bases. As of March 2025, TikTok recorded over 1 billion monthly active users with per-user monthly usage exceeding 46.54 hours, while Bilibili reached 220 million monthly active users with an average monthly usage of 16.42 hours.^[10]^ These metrics underscore the potential of short videos as powerful tools for public health communications.

Despite their advantages, significant concerns persist regarding the quality and reliability of the health information on these platforms. The absence of rigorous peer review and inconsistent regulatory oversight often results in variable content accuracy, with some videos containing misleading or incorrect information resulting in viewers’ misunderstanding of disease cognition, treatment methods, and health practices.^[11]^ Previous studies on conditions such as premature ovarian failure, hypertension, and thyroid eye disease treatment indicate that although health-related short videos frequently achieve high levels of user interaction, their educational quality and scientific credibility are often limited.^[12–14]^ Such discrepancies highlight a critical gap in the current digital health communication strategies. Therefore, a systematic evaluation of short videos pertaining to AML is essential to assess their informational quality, identify prevalent misconceptions, and inform future content regulations and designs.

This study aimed to analyze the content, quality, and reliability of AML-related short videos on TikTok and Bilibili. The findings may provide valuable references for improving patient education and public health information as well as offer guidance for the regulation of electronic health information dissemination.

## 2. Methods and materials

### 2.1. Ethical considerations

This study did not include clinical data, human specimens, or experimental animals. All materials were obtained from publicly available videos on TikTok and Bilibili with strict adherence to privacy protection principles. The data collection and analysis procedures fully complied with the platform terms and conditions. Since there was no direct contact with the users, ethical approval was not required.

### 2.2. The data sources and eligibility criteria

This study collected short videos related to “急性髓系白血病 (AML)” from Bilibili (https://www.bilibili.com) and TikTok (https://www.tiktok.com). To minimize bias from personalized recommendation algorithms, all searches were conducted via the web version without logging into accounts and in incognito mode to avoid interference from browsing history or algorithmic customization. The top 150 videos under the default comprehensive ranking on each platform were selected as a preliminary sample. The sample size of 150 videos per platform was selected based on methodologies commonly employed in prior cross-sectional studies evaluating health information on social media platforms,^[15,16]^ which typically analyze the top 100 to 200 search results to capture the content most readily accessible to users. This number represents a balance between comprehensively capturing the initial information landscape a typical user would encounter and maintaining feasibility for in-depth, manual quality assessment by trained evaluators. As the default ranking on these platforms is designed to surface the most popular and relevant content, analyzing the top-listed videos ensures the assessment of materials with the highest potential reach and public impact.

**Figure 1.**
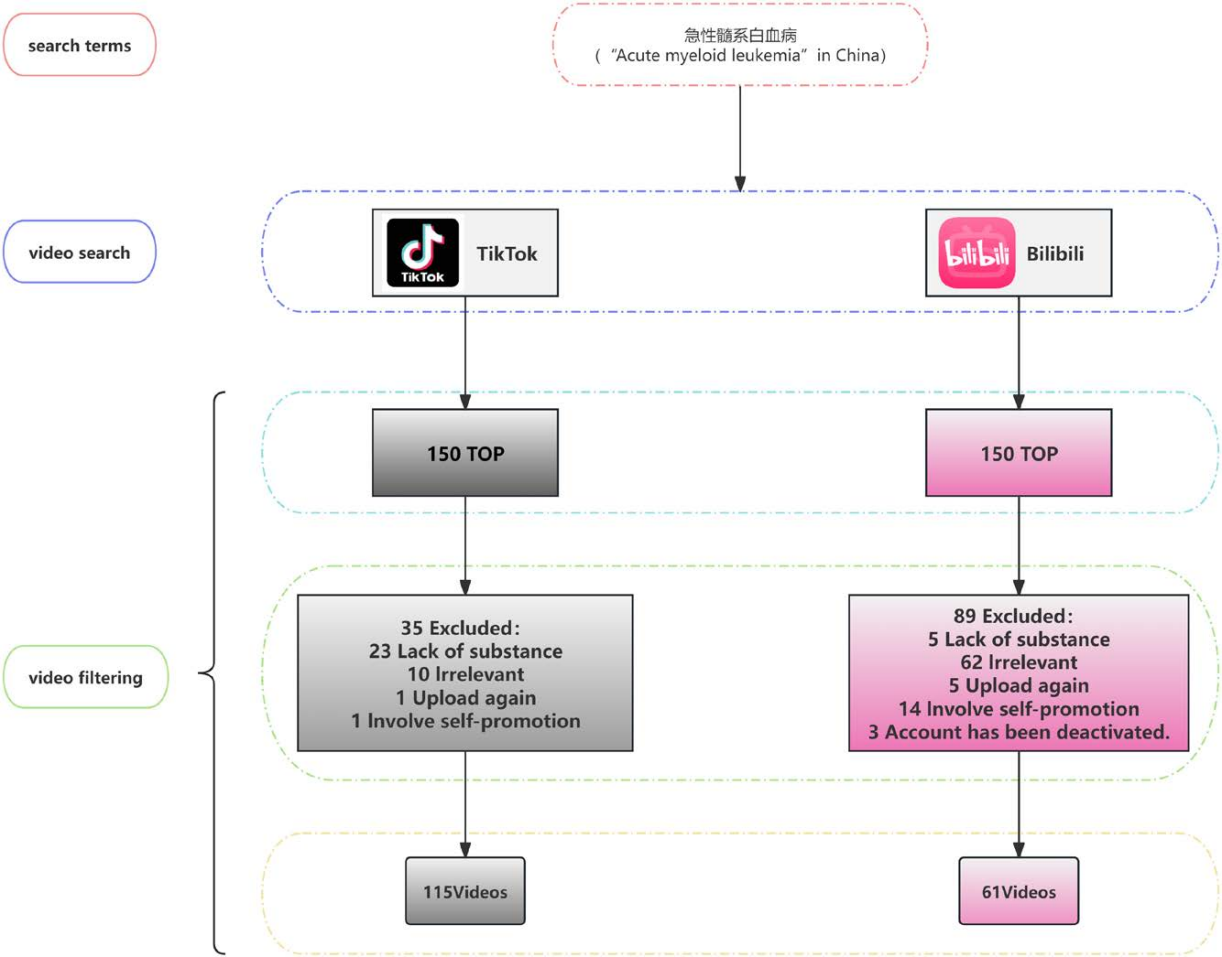
Flowchart illustrating the video selection process for analysis of AML information on TikTok and Bilibili. AML = acute myeloid leukemia.

The inclusion criteria were as follows: videos should be directly related to the pathogeny, typing, clinical manifestation, diagnosis, treatment, and prognosis of AML, and the content should be publicly accessible (private or restricted was excluded). The exclusion criteria were as follows: videos identified as advertising or promotional materials; content unrelated to AML (e.g., mentioning leukemia only metaphorically); videos from users who had logged out or accounts contained no substantive information; duplicate videos or multiple uploads of identical content, in which case only the earliest posted version was retained; and videos that became unavailable (deleted or private) during the data collection and analysis period. For each video, the following basic information was recorded: website URL, author information, number of likes, number of favorites, number of shares, number of comments, and video duration. The workflow of this study is illustrated in Figure 1.

### 2.3. Characteristics of the uploader

Video uploaders were divided into professionals and nonprofessionals. Professionals were further divided into hematologists and other specialists, whereas nonprofessionals were divided into patients and individual users.

### 2.4. Quality and reliability assessment

To evaluate the quality and reliability of short videos, this study used 3 scoring tools: global quality score (GQS) and modified DISCERN (mDISCERN) scores, and the *Journal of American Medical Association* (JAMA) benchmark criteria.^[17–19]^ The GQS evaluates online video quality using 5 levels, scoring from 1 (poor) to 5 (excellent). The mDISCERN is scored on a 5-point scale: clarity, relevance, traceability, robustness, and fairness. Each criterion was rated as “yes” (1 point) or “no” (0 points), with the cumulative score calculated (ranging from 0 to 5points). A higher score indicated greater reliability. The JAMA uses a benchmark of Authorship, Attribution, Currency, and Disclosure to produce a composite score ranging from 0 to 4, with higher scores indicating greater reliability and expertise. The specific evaluation items for these scales are presented in Tables 1–3. The evaluation was performed by 2 independent researchers (SY and YL) with relevant medical backgrounds who received unified training before the evaluation to ensure consistency and minimize bias. To ensure rigor of the study, a double-blind evaluation mechanism was used to ensure that the 2 researchers did not contact each other’s scores during the initial evaluation phase. In cases of disagreement, a third investigator was consulted to reach consensus. Furthermore, we used Cohen kappa (*k*)^[20]^ to quantify the agreement between the 2 raters. The *k* values were interpreted as follows: *k* > 0.8 indicated excellent consistency; 0.6 < *k* ≤ 0.8 suggested substantial agreement; 0.4 < *k* ≤ 0.6 signified moderate agreement; and *k* ≤ 0.4 was indicative of poor agreement.^[21]^ The analysis demonstrated excellent agreement, with *k* values of 0.923 for GQS, 0.906 for mDISCERN, and 0.928 for JAMA, all indicating excellent consistency.

**Table 1:** The global quality score quality criteria.

Item features	Points
Poor quality; poor flow of the videos; most information missing; not at all useful for patients	1
Generally poor quality; some information listed, but many important topics missing; of very limited use to patients	2
Moderate quality; suboptimal flow; some important adequately discussed, but other information poorly discussed; somewhat useful for patients	3
Good quality and generally good flow; most of the relevant information listed, but some topics not covered; useful for patients	4
Excellent quality and flow; very useful for patients	5

**Table 2:** The modified DISCERN quality criteria.

Reliability score
1. Is the video clear, concise, and understandable?
2. Are valid sources cited?
3. Is the content presented balanced and unbiased?
4. Are additional sources of content listed for patient reference?
5. Are areas of uncertainty mentioned?

1 point for answer “yes,” 0 point for answer “no”.

**Table 3:** The *Journal of the American Medical Association* benchmark criteria.

Score*	Score component	
1 score	Authorship	Author and contributor credentials and their affiliations should be provided.
1 score	Attribution	Clearly lists all copyright information and states references and sources for content.
1 score	Currency	Initial date of posted content and subsequent updates to content should be provided.
1 score	Disclosure	Conflicts of interest, funding, sponsorship, advertising, support, and video ownership should be fully disclosed.

* The criteria of each aspect were scored separately, and 1 point for each criterion with a total score of 4 points.

### 2.5. Statistical analysis

This study employed the Shapiro–Wilk test which was used to assess the normality of the variables extracted from the videos. For data with a parametric distribution, descriptive statistics were reported as the mean and standard deviation. Non-normally distributed data were summarized using median and interquartile range (IQR). The Mann–Whitney *U* test was used for nonparametric comparisons between 2 independent groups, whereas the Kruskal–Wallis H test was applied for comparisons among 3 or more groups. Spearman rank correlation analysis was applied to evaluate the association between general engagement metrics (including number of likes, favorites, shares, comments, and video duration) and video quality and reliability scores. All data processing and visualization were performed using the R software (https://www.r-project.org/) (version 4.3.3).

## 3. Results

### 3.1. Analysis of basic features of videos

A total of 176 videos were included in this study, and the detailed selection process is illustrated in Figure 1. Table 4 presents the characteristics of the included videos. In terms of platform distribution, TikTok contributed 115 videos, whereas Bilibili provided 61. On the TikTok platform (n = 115), AML-related short videos had a median duration of 80.00 seconds (IQR: 48.00–114.00). For user engagement metrics, the median number of likes was 175.00 (IQR: 80.50–380.00), the median number of comments was 34.00 (IQR: 11.50–132.50), the median number of shares was 30.00 (IQR: 7.00–72.50), and the median number of collections was 36.00 (IQR: 14.50–92.00). Quality assessment revealed a median GQS score of 3.00 (IQR: 2.00–4.00), median mDISCERN score of 2.00 (IQR: 1.00–3.00), and median JAMA score of 2.00 (IQR: 2.00–3.00).

**Table 4:** The general information, quality, and reliability scores of videos on TikTok and Bilibili.

Variables	Total (n = 176)	Bilibili (n = 61)	TikTok (n = 115)	*P*-value
Video length, M (Q_1_, Q_3_)	94.50 (55.00, 150.75)	155.00 (86.00, 619.00)	80.00 (48.00, 114.00)	<.001
Likes, M (Q_1_, Q_3_)	152.00 (51.50, 416.50)	52.00 (6.00, 548.00)	175.00 (80.50, 380.00)	.002
Collections, M (Q_1_, Q_3_)	32.00 (9.00, 129.25)	18.00 (2.00, 299.00)	36.00 (14.50, 92.00)	.095
Comments, M (Q_1_, Q_3_)	22.50 (5.00, 120.00)	6.00 (0.00, 93.00)	34.00 (11.50, 132.50)	<.001
Shares, M (Q_1_, Q_3_)	23.00 (3.50, 72.50)	8.50 (1.00, 70.75)	30.00 (7.00, 72.50)	.013
mDISCERN, M (Q_1_, Q_3_)	2.00 (1.00, 3.00)	2.00 (1.00, 3.00)	2.00 (1.00, 3.00)	.467
GQS, M (Q_1_, Q_3_)	3.00 (2.00, 4.00)	3.00 (2.00, 3.00)	3.00 (2.00, 4.00)	.744
JAMA1, M (Q_1_, Q_3_)	2.00 (2.00, 3.00)	2.00 (2.00, 3.00)	2.00 (2.00, 3.00)	.128

GQS = global quality score, JAMA = *Journal of American Medical Association*, mDISCERN = modified DISCERN.

On the Bilibili platform (n = 61), AML-related short videos had a median duration of 155.00 seconds (IQR: 86.00–619.00). For user engagement metrics, the median number of likes was 52.00 (IQR: 6.00–548.00), the median number of comments was 6.00 (IQR: 0.00–93.00), the median number of shares was 8.50 (IQR: 1.00–70.75), and the median number of collections was 18.00 (IQR: 2.00–299.00). Quality assessment revealed a median GQS score of 3.00 (IQR: 2.00–3.00), median mDISCERN score of 2.00 (IQR: 1.00–3.00), and median JAMA score of 2.00 (IQR: 2.00–3.00).

### 3.2. Video content

Table 5 provides a detailed comparison of the video content of TikTok and Bilibili.

**Table 5:** Comparison of video content on TikTok and Bilibili.

Variables	Total (n = 176)	Bilibili (n = 61)	TikTok (n = 115)	*P*-value
Typing, M (Q_1_, Q_3_)	0.00 (0.00, 1.00)	0.00 (0.00, 1.00)	0.00 (0.00, 1.00)	.014
Clinical manifestation, M (Q_1_, Q_3_)	0.00 (0.00, 1.00)	0.00 (0.00, 1.00)	0.00 (0.00, 0.00)	.002
Diagnosis, M (Q_1_, Q_3_)	0.00 (0.00, 1.00)	0.00 (0.00, 0.00)	0.00 (0.00, 1.00)	.069
Treatment, M (Q_1_, Q_3_)	1.00 (1.00, 1.00)	1.00 (1.00, 1.00)	1.00 (1.00, 1.00)	.111
Prognosis, M (Q_1_, Q_3_)	1.00 (0.00, 1.00)	0.00 (0.00, 1.00)	1.00 (0.00, 1.00)	<.001

**Figure 2.**
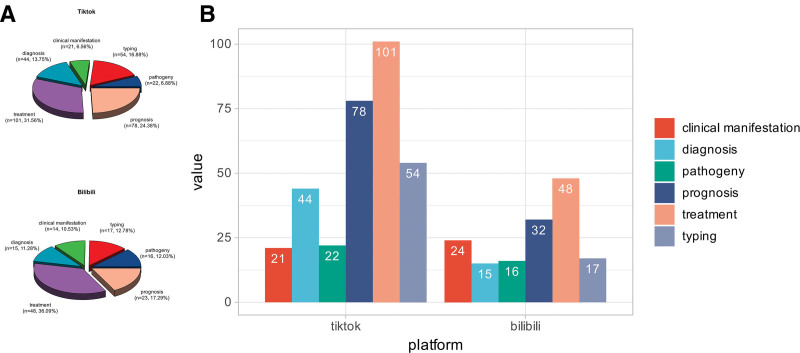
Comparative content assessment of AML videos between TikTok and Bilibili. (A) Content analysis. (B) Comparison of specific content quantities. AML = acute myeloid leukemia.

Figure 2A and B presents the content distribution of AML-related videos on TikTok and Bilibili. Analysis of video content revealed that themes related to “Treatment” (TikTok and Bilibili: n = 101, 31.6% and n = 48, 36.1%) and “Prognosis” (TikTok and Bilibili: n = 78, 24.4% and n = 23, 17.3%) were the most common. In contrast, content coverage related to “Pathogeny” and “Clinical Manifestation” was lacking.

### 3.3. Uploaders’ characteristics

**Figure 3.**
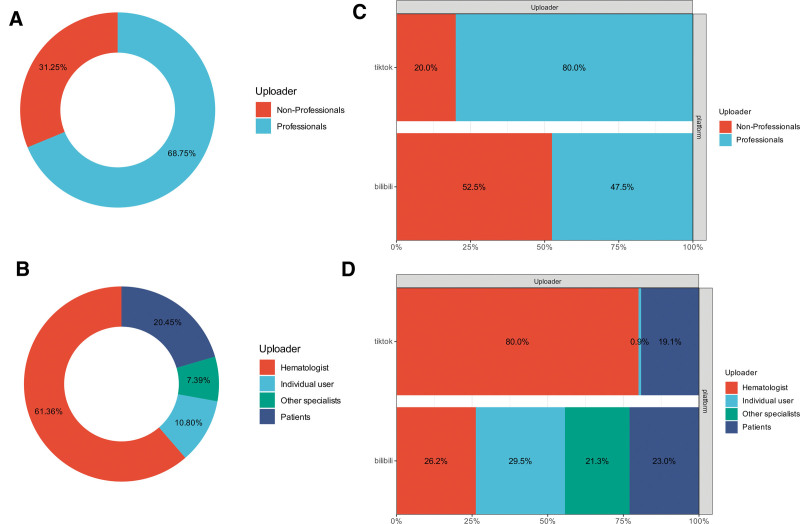
Distribution of video uploaders on Bilibili and TikTok. (A) Overall distribution of professional vs nonprofessional uploaders. (B) Overall distribution of video uploaders. (C) Distribution of professional vsnonprofessional uploaders on Bilibili and TikTok. (D) Distribution of video uploaders on Bilibili and TikTok.

As shown in Table 6, among all video uploaders, professionals accounted for 68.75% (n = 121), whereas nonprofessionals accounted for 31.25% (n = 55; Fig. 3A). Hematologists were the largest group (61.36%, n = 108), followed by patients (20.45%, n = 36), individual users (10.80%, n = 19), and other specialists (7.39%, n = 13; Fig. 3B).

**Table 6:** Comparison of video characteristics professionals versus nonprofessionals on TikTok and Bilibili.

Variables	Total (n = 176)	Nonprofessionals (n = 55)	Professionals (n = 121)	*P*-value
Video length, M (Q_1_, Q_3_)	94.50 (55.00, 150.75)	143.00 (96.00, 290.50)	80.00 (48.00, 119.00)	<.001
Likes, M (Q_1_, Q_3_)	152.00 (51.50, 416.50)	279.00 (59.50, 1505.50)	130.00 (49.00, 263.00)	.022
Collections, M (Q_1_, Q_3_)	32.00 (9.00, 129.25)	30.00 (7.50, 120.50)	32.00 (11.00, 128.00)	.968
Comments, M (Q_1_, Q_3_)	22.50 (5.00, 120.00)	93.00 (13.00, 437.00)	17.00 (4.00, 58.00)	<.001
Shares, M (Q_1_, Q_3_)	22.00 (3.00, 72.25)	27.00 (2.00, 73.50)	21.00 (4.00, 65.00)	.986
mDISCERN, M (Q_1_, Q_3_)	2.00 (1.00, 3.00)	1.00 (1.00, 2.00)	2.00 (2.00, 3.00)	<.001
GQS, M (Q_1_, Q_3_)	3.00 (2.00, 4.00)	2.00 (2.00, 3.00)	3.00 (2.00, 4.00)	<.001
JAMA1, M (Q_1_, Q_3_)	2.00 (2.00, 3.00)	1.00 (1.00, 2.00)	2.00 (2.00, 3.00)	<.001

GQS = global quality score, JAMA = *Journal of American Medical Association*, mDISCERN = modified DISCERN.

From the perspective of different platforms, TikTok content is primarily contributed by professionals, while on Bilibili, most content comes from nonprofessionals (Fig. 3C). A more detailed analysis revealed that hematologists were the largest group among uploaders on TikTok, whereas on Bilibili, the majority of content was contributed by individual users (Fig. 3D).

### 3.4. Comparison of AML short videos between platforms

**Figure 4.**
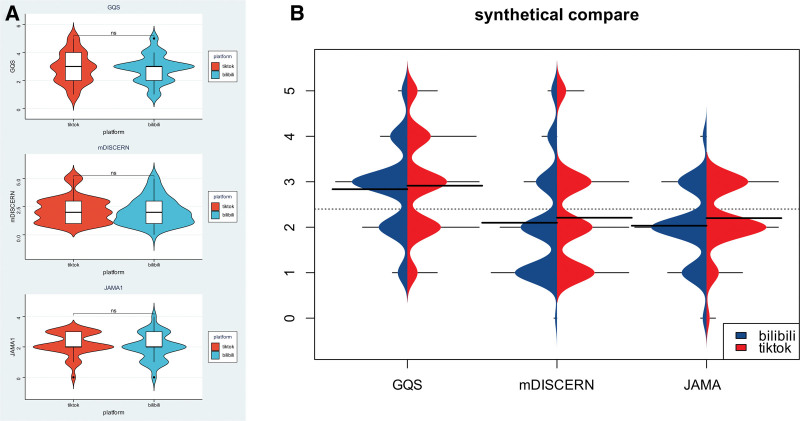
Comparison of quality and reliability scores between TikTok and Bilibili videos. (A) Comparison of specific scores. (b) Comparison of overall scores. GQS = global quality score, JAMA = *Journal of American Medical Association*, mDISCERN = modified DISCERN.

As shown in Table 4, the median video duration of the Bilibili platform was significantly longer than that of TikTok (155.00 vs 80.00 seconds, *P* < .001). However, TikTok videos performed better in terms of user interaction, with a markedly greater number of likes (175.00 vs 52.00, *P* = .002), comments (34.00 vs 6.00, *P* < .001), and shares (30.00 vs 8.50, *P* = .013). Crucially, there were no significant differences in the median GQS, mDISCERN, or JAMA scores between the 2 platforms (all *P* > .05; Fig. 4A and B).

### 3.5. Comparison of AML short videos among different uploaders (professionals vs nonprofessionals)

**Figure 5.**
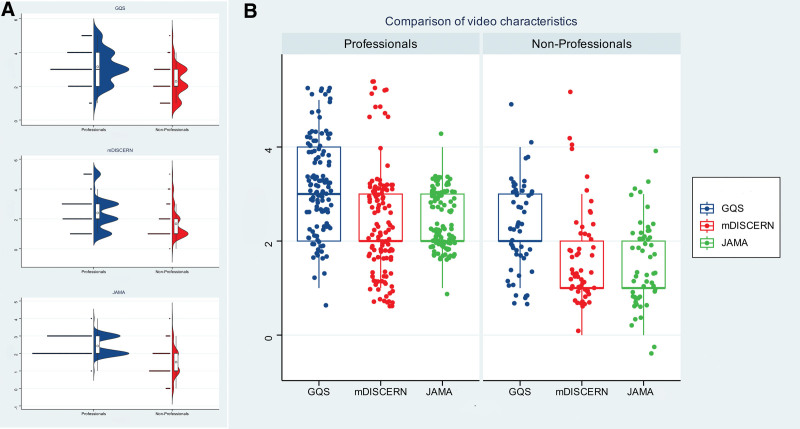
Distribution of quality and reliability scores across professionals versus nonprofessionals. (A) Comparison of specific scores. (B) Comparison of overall scores. GQS = global quality score, JAMA = *Journal of American Medical Association*, mDISCERN = modified DISCERN.

As shown in Figure 5A, significant differences were observed in various metrics of AML short videos between professionals and nonprofessionals. Furthermore, Figure 5B presents a clearer comparison of the overall quality scores between professionals and nonprofessionals. The professional group (n = 121) demonstrated significantly higher video quality scores than the nonprofessional group (n = 55), with statistically significant differences in GQS scores (3.00 vs 2.00, *P* < .001), mDISCERN scores (2.00 vs 1.00, *P* < .001), and JAMA scores (2.00 vs 1.00, *P* < .001; Table 6).

Videos from the nonprofessional group were significantly longer than those from the professional group (143.00 vs 80.00 minutes, *P* < .001). The user engagement metrics showed an interesting contrast: the nonprofessional group had significantly higher numbers of likes (279.00 vs 130.00, *P* = .022) and comments (93.00 vs 17.00, *P* < .001) compared with the professional group, while no significant differences were found in the number of shares (*P* = .986) and collections (*P* = .968) between the 2 groups (Table 6).

### 3.6. Comparison of AML short videos among different uploaders

**Figure 6.**
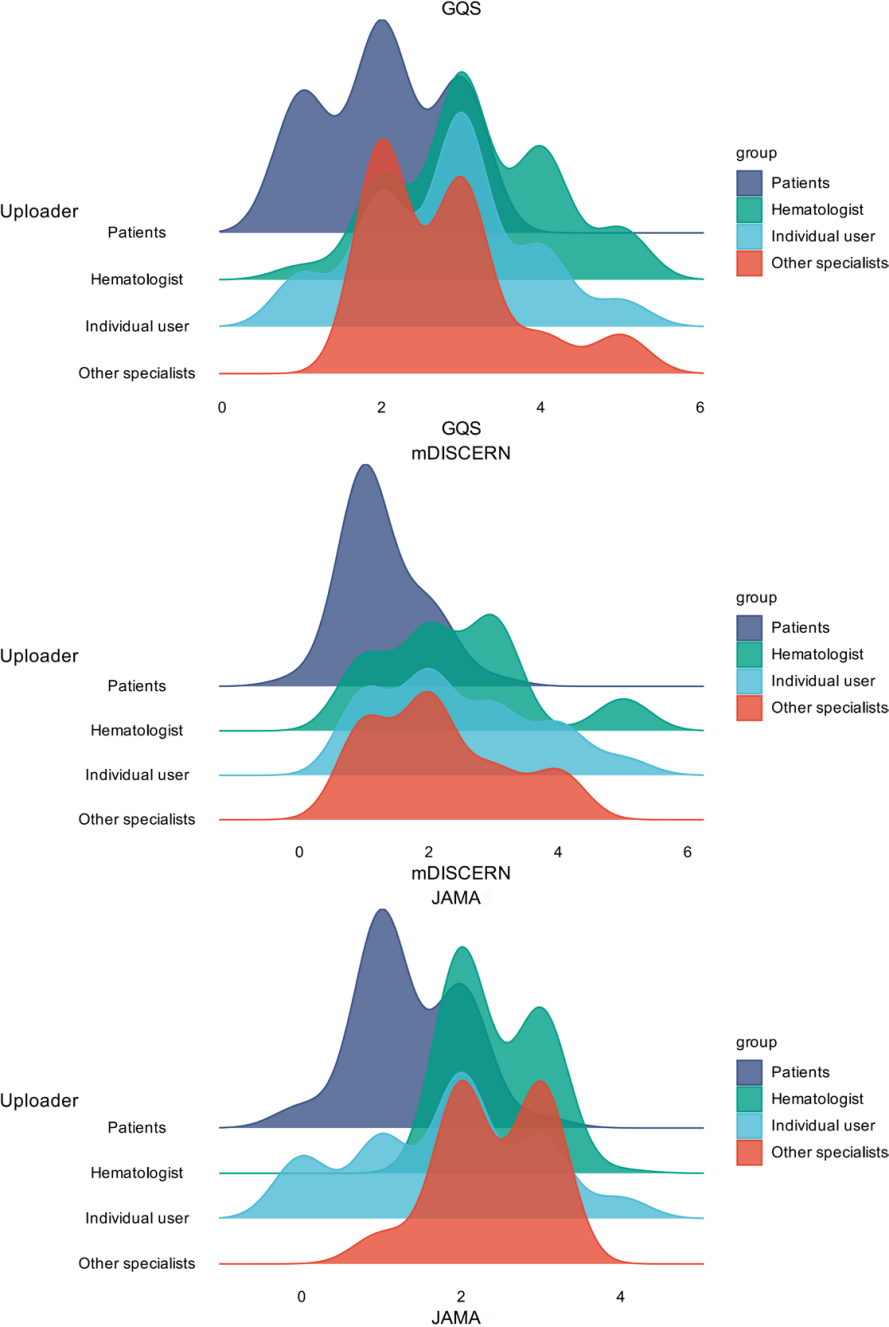
Distribution of quality and reliability scores across different types of uploader groups. GQS = global quality score, JAMA = *Journal of American Medical Association*, mDISCERN = modified DISCERN.

As shown in Table 7, among the different types of uploaders, there were significant differences in video length (*P* < .001), with individual users posting the longest videos. For user interaction, videos uploaded by the patients received the highest number of comments and likes (*P* < .001, *P* = .010). Most importantly, all information quality scores (GQS, mDISCERN, and JAMA) differed significantly among the uploaders (all *P* < .001). The content uploaded by hematologists received the highest scores, whereas content from patients received the lowest scores (Fig. 6). There were no significant differences in the number of collections or shares among the different upload types (*P* > .05).

**Table 7:** Characteristics of video uploaders about AML on TikTok and Bilibili.

Variables	Total (n = 176)	Hematologist (n = 108)	Individual user (n = 19)	Other specialists (n = 13)	Patients (n = 36)	*P*-value
Video length, M (Q_1_, Q_3_)	94.50 (55.00, 150.75)	77.50 (49.50,109.75)	196.00 (104.50,610.00)	86.00 (44.00,182.00)	120.50 (91.50,204.50)	<.001
Likes, M (Q_1_, Q_3_)	152.00 (51.50, 416.50)	142.00 (59.25,260.75)	85.00 (11.00,1832.50)	19.00 (5.00,411.00)	352.00 (80.00,1281.50)	.010
Collections, M (Q_1_, Q_3_)	32.00 (9.00, 129.25)	35.00 (13.00,105.25)	30.00 (4.00,201.00)	17.00 (0.00,823.00)	31.00 (8.75,106.25)	.893
Comments, M (Q_1_, Q_3_)	22.50 (5.00, 120.00)	17.00 (6.75,58.75)	6.00 (0.50,91.50)	3.00 (0.00,28.00)	165.00 (53.75,599.25)	<.001
Shares, M (Q_1_, Q_3_)	22.00 (3.00, 72.25)	23.50 (6.00,62.00)	24.00 (1.00,90.50)	2.00 (1.00,189.00)	31.00 (3.00,73.25)	.498
mDISCERN, M (Q_1_, Q_3_)	2.00 (1.00, 3.00)	2.00 (2.00,3.00)	2.00 (1.50,3.00)	2.00 (1.00,3.00)	1.00 (1.00,2.00)	<.001
GQS, M (Q_1_, Q_3_)	3.00 (2.00, 4.00)	3.00 (3.00,4.00)	3.00 (2.00,3.00)	3.00 (2.00,3.00)	2.00 (1.00,3.00)	<.001
JAMA1, M (Q_1_, Q_3_)	2.00 (2.00, 3.00)	2.00 (2.00,3.00)	2.00 (1.00,2.50)	2.00 (2.00,3.00)	1.00 (1.00,2.00)	<.001

AML = acute myeloid leukemia, GQS = global quality score, JAMA = *Journal of American Medical Association*, mDISCERN = modified DISCERN.

### 3.7. Correlation analysis

**Figure 7.**
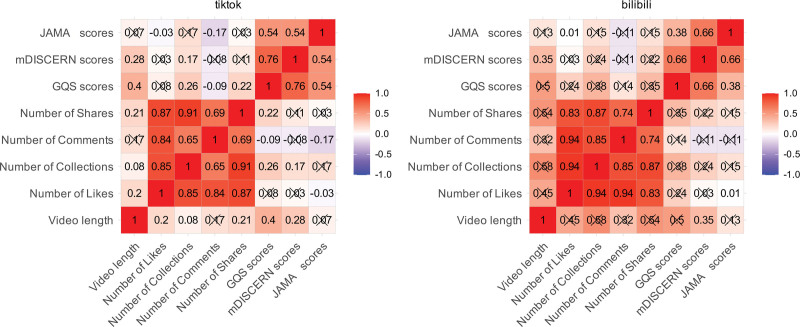
Correlation matrix of video engagement metrics and quality scores on TikTok and Bilibili. GQS = global quality score, JAMA = *Journal of American Medical Association*, mDISCERN = modified DISCERN.

Spearman correlation analysis was conducted to evaluate the relationships between video interaction metrics (likes, comments, collections, and shares) and quality assessment scores (GQS, mDISCERN, and JAMA). As shown in Figure 7, a significant positive correlation was observed between the interaction metrics. Specifically, for TikTok, strong and statistically significant correlations were found between likes and collections (*R* = 0.85, *P* < .001), likes and comments (*R* = 0.84, *P* < .001), and likes and shares (*R* = 0.87, *P* < .001). Similarly, for Bilibili, correlations were also strong between likes and collections (*R* = 0.94, *P* < .001), likes and comments (*R* = 0.94, *P* < .001), and likes and shares (*R* = 0.83, *P* < .001). In contrast, no significant correlation was found between any of the information quality scores (GQS, mDISCERN, and JAMA) and user interaction metrics (e.g., collections, comments, and shares; all *P* > .05).

## 4. Discussion

### 4.1. Research background

The rapid development of short video platforms has made social media an important source of public access to health-related information. The public pays more attention to disease treatment methods and management suggestions, while videos involving professional content, such as pathological mechanisms, require the support of professional medical knowledge. Ordinary viewers who lack a medical background often find it difficult to understand such content and are naturally less willing to watch it. In addition, AML treatment has rapidly evolved in recent years, with the approval of several novel and targeted agents. Despite these advances, secondary AML remains an area of clear unmet need, with poor response to current standard regimens and poor prognosis,^[22]^ which means there is a greater need for the opposite. With the rise of social media, especially short video platforms, the way the public accesses health information has undergone a significant transformation.^[23,24]^ TikTok and Bilibili have become major channels for the dissemination of health-related content, attracting a large user base.^[25,26]^ Assessment of the health information quality and reliability is very important. Previous studies have shown that the popularization of health knowledge to patients with acute leukemia in clinical medicine can help patients better familiarize themselves with the diseases and enhance their own awareness and ability to protect more.^[27]^

This study evaluated the ecology of AML-related content on short video platforms and revealed the complex relationship between platform characteristics, uploaders’ identity, and content quality. These findings have important implications for the theory and practice of digital health communications.

### 4.2. Advantages of high user engagement

This study shows that content on TikTok and videos created by patients received significantly higher user engagement (median likes: TikTok M = 175.00 vs Bilibili M = 52.00; median comments for patient videos: M = 165.00). This finding corroborates the unique value of social media in health communication: it provides emotional support and a sense of community for patients. Research by Farsi et al^[28]^ indicates that social media can effectively alleviate psychological pressure and social isolation in patients with chronic diseases through peer support. High engagement reflects the content’s ability to resonate with viewers, facilitating experience-sharing and emotional exchange among patients. This social media-based support system provides an important channel for emotional expressions. Further studies by Devan et al^[29]^ confirmed the unique value of patient narratives in providing emotional support and sharing experiences in health communication.

### 4.3. The 1-sidedness of content coverage and the lack of systematic health education

Although treatment and prognosis are the most important topics for patients, the coverage of basic medical knowledge such as etiology, classification, and diagnosis is seriously insufficient, reflecting the fragmentation and 1-sided nature of the current short video content ecology. Although Bilibili is better than TikTok in the coverage of some medical topics, it still lacks comprehensive and systematic education on AML. This content ecology may make the public unable to form a complete cognition of the disease –to focused on the treatment outcome – and ignore the importance of early diagnosis and standardized diagnosis and treatment, which is not conducive to the primary and secondary prevention of the disease.^[30]^

Of particular concern is insufficient public awareness regarding the etiology, classification, and early clinical manifestations of AML, which frequently contributes to delays in diagnosis, thereby elevating disease burden and worsening outcomes. Without timely intervention, patients face a high risk of severe complications, including life-threatening infections due to neutropenia, significant hemorrhage resulting from thrombocytopenia, symptomatic anemia, and tumor lysis syndrome associated with rapid leukemic cell proliferation.^[31]^ Furthermore, disease progression can lead to multi-organ failure, substantially increasing treatment difficulty and mortality, and placing considerable physical, emotional, and socioeconomic strain on patients, caregivers, and health systems.^[32]^

To mitigate these issues, it is critical to improve the accuracy, depth, and reach of AML-related educational content, especially regarding pathogeny, typing, and diagnosis. Public health initiatives and digital platforms should facilitate the distribution of validated and clearly structured information to enhance health literacy.^[33]^ Improved public understanding can promote earlier recognition of warning signs and encourage the timely seeking of medical care. Ultimately, these measures can help reduce the overall burden of AML through more effective prevention and earlier treatment initiation.^[34]^

### 4.4. The phenomenon and risk of “quality–quantity separation” between information quality and user interaction

The core finding of this study is that it reveals a significant “quality–quantity separation” in AML information communication (i.e., high interactivity does not equal high information quality), which echoes the conclusion of an earlier study on YouTube by Madathil et al.^[35]^ On the one hand, our findings suggest that professionals (especially hematologists) are likely the most reliable source^[36]^ of high-quality, evidence-based information. Their content includes accuracy, balance, and reliability and is the cornerstone of building public health information infrastructure. On the other hand, lay people (especially patients), the process of sharing, constructing, and listening to similar others through stories, elicits feelings of empowerment and empathy and strengthens social connection.^[37]^ More can stimulate the resonance and interaction between users. However, such content is also more likely to contain inaccurate medical information, 1-sided descriptions of treatment effects, or unproven alternative therapies,^[38]^ posing a potential risk to users with a limited ability to discriminate information. This disconnection poses a central challenge to public health information literacy.

### 4.5. Platform algorithms and subculture shaping the mode of health information dissemination

This study observed that TikTok has a significant advantage in the user interaction index and is designed to maximize user engagement and retention time is highly recommended algorithm.^[39]^ Its “short and fast” and highly entertaining content characteristics are more likely to trigger instant viral transmission. In contrast, Bilibili users have a higher acceptance of medium- and long-form and in-depth content, which is related to their community culture and user structure. However, the key finding is that although platform characteristics cause a wide range of dissemination power, they do not lead to a fundamental difference in the objective quality of the content. This may suggest that the scientific rigor of the information is relatively independent of the platform, which is more likely to depend on other factors. This suggests that when assessing health information ecology, entrepreneurship and “information quality” should be considered as 2 independent and potentially divergent dimensions. However, these interpretations regarding platform algorithms and subculture are hypothesis-generating, and based on our observations in the context of existing literature, they need further investigation through dedicated studies.

### 4.6. Based on the above findings, we propose recommendations

For the public and patients, health literacy^[40]^ should be promoted, information sources should be actively identified, and social media should be viewed as a supplementary channel for initial information, emotional support, and peer communication, rather than as a basis for clinical decision-making.

Healthcare professionals should actively engage in new media platforms and learn to translate complex medical knowledge into short video content that is both rigorous and engaging. Science communicators should adhere to high academic standards, cite authoritative references, and clearly communicate any uncertainties^[41]^ in existing knowledge for effective knowledge translation.^[42]^ Take the initiative to occupy the position of communication, especially in the etiology, diagnosis, and other weak links of current knowledge.

Platforms and regulators should improve the content review mechanism of AML-related videos, strictly limit advertising placement,^[43]^ correctly disseminate effective health information, curb the spread^[44]^ of false information, develop algorithmic tools to prioritize high-quality content, strengthen the review of obvious false information, fulfill corporate social responsibility, and optimize the platform information ecology.

## 5. Limitations

This study had several limitations. First, it focuses only on TikTok and Bilibili, which, although dominant in the Chinese market, may limit the generalizability of the findings to other social media platforms or audiences in different cultural contexts. Second, given the rapid update cycle of short videos, results may not fully capture the latest trends and developments. Third, the relatively small sample size may have affected the representativeness and statistical power of the findings, and future studies should expand the sample size to strengthen the robustness of the conclusions. Fourth, although validated instruments were employed, the evaluation process inevitably involved a degree of subjectivity, which may have introduced an assessor bias. Fourth, it is also important to note that platform algorithms are dynamic and personalized. Although searches were conducted in incognito mode, our results could still be influenced by factors such as geographic location and the timing of data collection. Our findings thus represent a specific snapshot and may not fully generalize to all users or to searches conducted at different times.

## 6. Conclusion

This study employed 3 validated tools to systematically evaluate the quality and user engagement of short AML-related videos on TikTok and Bilibili. The results indicate that social media has become an important channel for the public to access health information on AML; however, the overall quality and reliability of such content remains moderate, with videos uploaded by hematologists achieving the highest ratings. The content structure was found to be incomplete, particularly with insufficient coverage for diagnosis and prevention. In the future, hematologists should be encouraged to actively contribute to AML science communication, while platforms should strengthen regulations and algorithmic optimization to promote the dissemination of high-quality AML short videos, thereby enhancing public health literacy and supporting disease prevention.

## Acknowledgments

The authors express their gratitude for this support.

## Author contributions

**Conceptualization:** Siqin Yang.

**Data curation:** Siqin Yang.

**Formal analysis:** Siqin Yang.

**Investigation:** Yuqing Li.

**Methodology:** Siqin Yang.

**Project administration:** Jun Tao.

**Supervision:** Jun Tao.

**Writing – original draft:** Siqin Yang.

**Writing – review & editing:** Yuqing Li.

## References

[1] Venugopal S Sekeres MA. Contemporary management of acute myeloid leukemia: a review. JAMA Oncol. 2024;10:1417–25. 39115831 10.1001/jamaoncol.2024.2662

[2] Siegel RL Miller KD Wagle NS Jemal A. Cancer statistics, 2023. CA Cancer J Clin. 2023;73:17–48. 36633525 10.3322/caac.21763

[3] Siegel RL Kratzer TB Giaquinto AN Sung H Jemal A. Cancer statistics, 2025. CA Cancer J Clin. 2025;75:10–45. 39817679 10.3322/caac.21871 PMC11745215

[4] Ning L Li D Lu P Que Y. Exploring the determinants that influence hospital costs of induction therapy for acute myeloid leukemia. Leuk Lymphoma. 2021;62:1211–8. 33300383 10.1080/10428194.2020.1855339

[5] Buckley SA Jimenez-Sahagun D Othus M Walter RB Lee SJ. Quality of life from the perspective of the patient with acute myeloid leukemia. Cancer. 2018;124:145–52. 28881384 10.1002/cncr.30982 PMC5749925

[6] Brownstein CG Twomey R Temesi J. Physiological and psychosocial correlates of cancer-related fatigue. J Cancer Surviv. 2022;16:1339–54. 34609702 10.1007/s11764-021-01115-6

[7] Sørensen HL Schjølberg TK Småstuen MC Utne I. Social support in early-stage breast cancer patients with fatigue. BMC Womens Health. 2020;20:243. 33121476 10.1186/s12905-020-01106-2 PMC7599095

[8] Mirkovski K Gaskin JE Hull DM Lowry PB. Visual storytelling for improving the comprehension and utility in disseminating information systems research: evidence from a quasi-experiment. Inf Syst J. 2019;29:1153–77.

[9] China Internet Network Information Center (CNNIC). The 55th statistical report on China’s internet development. 2025 [cited January 2025]; https://www.cnnic.com.cn/IDR/ReportDownloads/202505/P020250514564119130448.pdf. Accessed 15 August 2025.

[10] QuestMobile. QuestMobile 2025 panoramic ecological traffic spring report. 2025 [cited August 3, 2025]; https://www.questmobile.com.cn/research/report/1917055006613278722. Accessed 15 August 2015.

[11] Memioglu T Ozyasar M. Analysis of YouTube videos as a source of information for myocarditis during the COVID-19 pandemic. Clin Res Cardiol. 2022;111:1113–20. 35471259 10.1007/s00392-022-02026-x PMC9039268

[12] Wu J Wu G Che X Guo J. The quality and reliability of short videos about hypertension on TikTok: a cross-sectional study. Sci Rep. 2025;15:25042. 40646027 10.1038/s41598-025-08680-1 PMC12254506

[13] Wang H Zhang H Cao J Zhang F Xiong W. Quality and content evaluation of thyroid eye disease treatment information on TikTok and Bilibili. Sci Rep. 2025;15:25134. 40646154 10.1038/s41598-025-11147-y PMC12254225

[14] Xu R Ren Y Li X Su L Su J. The quality and reliability of short videos about premature ovarian failure on Bilibili and TikTok: cross-sectional study. Digit Health. 2025;11:20552076251351077. 40534890 10.1177/20552076251351077 PMC12174690

[15] Chen Y Wang Q Huang X. The quality and reliability of short videos about thyroid nodules on BiliBili and TikTok: cross-sectional study. Digit Health. 2024;10:1338–48. 10.1177/20552076241288831 PMC11459542 39381823

[16] Gong X Chen M Ning L Zeng L Dong B. The quality of short videos as a source of coronary heart disease information on TikTok: cross-sectional study. JMIR Form Res. 2024;8:e51513. 39226540 10.2196/51513 PMC11408897

[17] Bernard A Langille M Hughes S Rose C Leddin D Veldhuyzen van Zanten S. A systematic review of patient inflammatory bowel disease information resources on the World Wide Web. Am J Gastroenterol. 2007;102:2070–7. 17511753 10.1111/j.1572-0241.2007.01325.x

[18] Kanzow P Büttcher AF Wiegand A Schwendicke F. Quality of information regarding repair restorations on dentist websites: systematic search and analysis. J Med Internet Res. 2020;22:e17250. 32062595 10.2196/17250 PMC7191344

[19] Silberg WM Lundberg GD Musacchio RA. Assessing, controlling, and assuring the quality of medical information on the internet: caveant lector et viewor–Let the reader and viewer beware. JAMA. 1997;277:1244–5. 9103351

[20] Cohen J. A coefficient of agreement for nominal scales. Educ Psychol Meas. 1960;20:37–46.

[21] Landis JR Koch GG. The measurement of observer agreement for categorical data. Biometrics. 1977;33:159–74. 843571

[22] Abaza Y Mcmahon C Garcia JS. Advancements and challenges in the treatment of AML. Am Soc Clin Oncol Educ Book. 2024;44:e438662. 38662975 10.1200/EDBK_438662

[23] Liu Q Zheng Z Zheng J. Health communication through news media during the early stage of the COVID-19 outbreak in China: digital topic modeling approach. J Med Internet Res. 2020;22:e19118. 32302966 10.2196/19118 PMC7189789

[24] Yuan Y Wang Q. Characteristics, hotspots, and prospects of short video research: a review of papers published in China from 2012 to 2022. Heliyon. 2024;10:e24885. 38318019 10.1016/j.heliyon.2024.e24885 PMC10839982

[25] Liang J Wang L Song S. Quality and audience engagement of Takotsubo syndrome-related videos on TikTok: content analysis. J Med Internet Res. 2022;24:e39360. 36155486 10.2196/39360 PMC9555329

[26] Qi Y Han J Lu X Wang Z Ren H Zhang X. A study on satisfaction evaluation of Chinese mainstream short video platforms based on grounded theory and CRITIC-VIKOR. Heliyon. 2024;10:e30050. 38707463 10.1016/j.heliyon.2024.e30050 PMC11068598

[27] Dedoncker A Lejeune C Dupont C. Nurse-led educative consultation setting personalized tertiary prevention goals after cardiovascular rehabilitation: evaluation of patient satisfaction and long-term effects. Rehabil Nurs. 2012;37:105–13. 22549627 10.1002/RNJ.00042

[28] Farsi D Martinez-Menchaca HR Ahmed M Farsi N. Social media and health care (part II): narrative review of social media use by patients. J Med Internet Res. 2022;24:e30379. 34994706 10.2196/30379 PMC8783277

[29] Devan H Elphick-laveta T Lynch M. “Power of storytelling”: a content analysis of chronic pain narratives on YouTube. Can J Pain. 2021;5:117–29.

[30] Frey E Bonfiglioli C Brunner M Frawley J. Parents’ use of social media as a health information source for their children: a scoping review. Acad Pediatr. 2022;22:526–39. 34906742 10.1016/j.acap.2021.12.006

[31] Döhner H Weisdorf DJ Bloomfield CD. Acute myeloid leukemia. N Engl J Med. 2015;373:1136–52. 26376137 10.1056/NEJMra1406184

[32] Shallis RM Wang R Davidoff A Ma X Zeidan AM. Epidemiology of acute myeloid leukemia: recent progress and enduring challenges. Blood Rev. 2019;36:70–87. 31101526 10.1016/j.blre.2019.04.005

[33] Stellefson M Paige SR Chaney BH Chaney JD. Evolving role of social media in health promotion: updated responsibilities for health education specialists. Int J Environ Res Public Health. 2020;17:1153. 32059561 10.3390/ijerph17041153 PMC7068576

[34] Dizon DS Kamal AH. Cancer statistics 2024: all hands on deck. CA Cancer J Clin. 2024;74:8–9. 38230825 10.3322/caac.21824

[35] Madathil KC Rivera-Rodriguez AJ Greenstein JS Gramopadhye AK. Healthcare information on YouTube: a systematic review. Health Informatics J. 2014;21:173–94. 24670899 10.1177/1460458213512220

[36] Chou WYS Oh A Klein WMP. Addressing health-related misinformation on social media. JAMA. 2018;320:2417–8. 30428002 10.1001/jama.2018.16865

[37] Briant KJ Halter A Marchello N Escareño M Thompson B. The power of digital storytelling as a culturally relevant health promotion tool. Health Promot Pract. 2016;17:793–801. 27402721 10.1177/1524839916658023 PMC5065376

[38] Suarez-Lledo V Alvarez-Galvez J. Prevalence of health misinformation on social media: systematic review. J Med Internet Res. 2021;23:e17187. 33470931 10.2196/17187 PMC7857950

[39] Klug D Qin Y Evans M Kaufman G. Trick and please. A mixed-method study on user assumptions about the TikTok algorithm. In: Proceedings of the 13th ACM Web Science Conference（Virtual Event, United Kingdom). 2021:84–92.

[40] Estrela M Semedo G Roque F Ferreira PL Herdeiro MT. Sociodemographic determinants of digital health literacy: a systematic review and meta-analysis. Int J Med Inform. 2023;177:105124. 37329766 10.1016/j.ijmedinf.2023.105124

[41] Kanchan S Gaidhane A. Social media role and its impact on public health: a narrative review. Cureus. 2023;15:e33737. 36793805 10.7759/cureus.33737 PMC9925030

[42] Van Kessel R Wong BLH Clemens T Brand H. Digital health literacy as a super determinant of health: more than simply the sum of its parts. Internet Interv. 2022;27:100500. 35242586 10.1016/j.invent.2022.100500 PMC8861384

[43] Yeung A Ng E Abi-Jaoude E. TikTok and attention-deficit/hyperactivity disorder: a cross-sectional study of social media content quality. Can J Psychiatry. 2022;67:899–906. 35196157 10.1177/07067437221082854 PMC9659797

[44] Jancey J Leaver T Wolf K. Promotion of e-cigarettes on TikTok and regulatory considerations. Int J Environ Res Public Health. 2023;20:5761. 37239490 10.3390/ijerph20105761 PMC10217796

